# COVID-19 Life Events-Anxiety Inventory (C-19LAI): development, reliability, and validity study on Egyptian population

**DOI:** 10.1186/s43045-021-00101-z

**Published:** 2021-04-02

**Authors:** Omaima Refat Elsayed Madkor, Khalid E. Elsorady, Dina H. Abdelhady, Passant AbdulJawad, Dina Aly El Gabry

**Affiliations:** 1 The Martyr Engineer Ahmed Shawky Hospital for Elderly Medicine, Ain Shams University Hospitals, Cairo, Egypt; 2 Department of Geriatrics and Gerontology, Faculty of Medicine, Ain Shams University, Cairo, Egypt; 3 Department of Statistics, Faculty of Commerce, Tanta University, Tanta, Egypt; 4 Department of Psychology, Faculty of Art, Kafrelsheikh University, Kafr El Sheikh, Egypt; 5 Scientific Research Development Unit, Tanta University, Tanta, Egypt; 6 Neuropsychiatry Department, Okasha Institute of Psychiatry, Ain Shams University, Abbasiya, Cairo, 1156 Egypt

**Keywords:** COVID-19, Life Events-Anxiety Inventory (C-19LAI), Anxiety, Life events, Pandemic

## Abstract

**Background:**

The COVID-19 Life Events-Anxiety Inventory (C-19LAI) is a newly developed tool and the only Arabic tool for assessing and measuring anxiety related to different life events during the COVID-19 pandemic. The aim of the study was to test the validity and reliability of this newly designed tool. We used a cross sectional validation multiphasic study and applied the tool on 500 subjects together with the State-Trait Anxiety Inventory (STAI).

**Results:**

The COVID-19 Life Events-Anxiety Inventory (C-19LAI) showed validity of 73.6% and sensitivity of 85.2%, with acceptable reliability of *α* = 0.815 and 0.947, respectively. The Life Events Scale and Anxiety Scale of the C-19LAI correlated significantly (*p* ≤ 0.01) with the State-Trait Anxiety Inventory (*r* = 0.289 and *r* = 0.407, respectively).

**Conclusion:**

The COVID-19 Life Events-Anxiety Inventory (C-19LAI) Scale is a reliable and valid scale that can measure anxiety and events related to anxiety during the COVID 19 pandemic.

**Supplementary Information:**

The online version contains supplementary material available at 10.1186/s43045-021-00101-z.

## Background

On December 31, 2019, the World Health Organization (WHO) received reports of several cases of viral pneumonia of unknown cause in Wuhan, China [1]. The rapid spread of the infection in China created a major health problem. The lockdown in many cities and shortage of health care facilities increased people’s risk of anxiety and depression [2]. On February 11, 2020, the International Committee on Taxonomy of Viruses named the new virus “severe acute respiratory syndrome coronavirus 2 (SARS-CoV-2)” and the WHO designated “COVID-19” as the name of this new disease [3] and declared it a pandemic on March 11, 2020 [4]. Over 25 million cases of COVID-19 have been reported globally, resulting in more than 843,000 deaths as of August 30, 2020 [5]. The COVID-19 pandemic is associated with a psychological crisis with relatively little psychological support for those who are affected, which has had a significant effect on their daily lives [6] and has increased the risk of post-traumatic stress and health anxiety [7–10].

Anxiety occurs when the autonomic nervous system is activated by subjective feelings of tension and nervousness [11]. Excessive health anxiety could lead individuals to misinterpret different symptoms as COVID-19 [9, 12] with subsequent increased risk of anxiety, depression, and even suicide as previously reported in India [13]. The unknown nature of the virus and excessive exposure to media, quarantine, and isolation may induce further psychological harm, fear, stress, and anxiety [14–16]. This has negatively affected public health [6] and different sectors in the community through the viral spread and increased risk of mortality [17–19].

Adherence to certain health advices such as stay at home in order to decrease viral transmission could also increase the risk of anxiety [20]. This could, in turn, weaken the immune system and increase the risk of infection [21]. The success of various public health strategies such as vaccination and social distancing is related to psychological factors [22]. Healthily coping with stress is essential for resilience [23]. In addition, cognitive–behavioral interventions could reduce health anxiety toward COVID-19 [24].

Anxiety can be assessed using both old and newer disease-specific tools such as the State-Trait Anxiety Inventory (STAI) for adults [25] and Coronavirus Anxiety Scale (CAS). STAI is a standardized questionnaire developed in 1983 by Charles D. Spielberger [26–28] and has been used in both research and clinical domains to study the effect of stress and anxiety and its role in performance and learning. Trait anxiety (T-Anxiety) and state anxiety (S-Anxiety) are similar to potential and kinetic energy, respectively. In other words, T-Anxiety refers to anxiety proneness to a stressful situation and could predict the intensity of their S-Anxiety reactions in the future depending on previous experiences of different persons [25]. On the other hand, various new public health measures have been implemented recently in order to assess the effects of the COVID-19 pandemic on daily life and behavior all over the world such as the newly designed CAS, which is comparable to the Generalized Anxiety Disorder-7 scale for identifying dysfunctional anxiety associated with the COVID-19 pandemic [29]. Our aim was to design a tool that helps enhancing persons and community awareness of events in individual’s life that might happen during COVID-19 pandemics and might have a negative impact on mental health especially anxiety, to facilitate provocative engagement in disaster risk reduction activities. In addition to a quantitative anxiety scale that could be used as an outcome measure with established reliability and validity for use in clinical trials and interventions during pandemics, an understanding of the relationship between life events and anxiety is important for policymakers as this might reduce the rising cost of mental health care at the same time it is very important for social and mental health professionals.

## Methods

This is a cross sectional validation multiphasic study aimed to test validity and reliability of the COVID-19 Life Events-Anxiety Inventory (C-19LAI) during the current COVID-19 pandemic in the Egyptian community using a web-based survey to settle novel inventory with acceptable validity and reliability for assessment of COVID-19 related anxiety among different sectors of population. Only for illiterate people or people who are not available online, participants were recruited from community to settle a battery of life events and anxiety related with COVID-19. COVID-19 life events means distressing events that took place during or as a result of the current COVID-19 pandemic that might have a negative impact on mental health by establishing anxiety that can take place as an example being personally diagnosed with coronavirus or having family member or a friend diagnosed with COVID-19, death of a family member or a friend, exhaustion due to quarantine, health precautions and instructions related to COVID-19 pandemic, fear of getting medical consultation, and of course financial, educational, and family and marriage problems that takes place as a consequence to the current pandemic.

### Participants

A total of 500 subjects were recruited from the community through convenience sampling and their demographic data are reported in Table 1. All calculations were performed at 95% confidence interval, 0.80 power of the study, and *α* error of 0.05. We included both males and females. All participants provided signed informed consent following a full explanation of the study. Participation was voluntary and patients had the right to withdraw at any time without giving a reason. Inclusion criteria were male and females, aged 18 years or older, and willing to participate. Demographical characteristics of the sample are shown in Table 1.

**Table 1:** Demographics of study participants

Variables	*N*	%
**Sex**		
Male	182	36.4
Female	318	63.6
**Age (years)**		
Less than 40	260	52.0
40-59	170	34.0
60 and over (60-86 years)	70	14.0
**Marital status**		
Widowed	35	7.0
Single	141	28.2
Married	300	60.0
Separated/divorced	24	4.8
**Educational level**		
Less than 12 years	114	22.8
More than 12 years	386	77.2
**Governorate**		
Cairo	158	31.6
Gharbia	116	23.2
Giza	85	17.0
Other	141	28.2
**Residence**		
Urban	443	88.6
Rural	57	11.4
**Monthly income**		
On financial support	92	18.4
Only basic needs	227	45.4
More than just basic needs	181	36.2
**Employment status**		
Employed	129	25.8
Unemployed	294	58.8
**Occupational level**		
Non skilled	124	24.8
Owners of medium shops	51	10.2
Clerical support workers	4	.8
Skilled and semi-skilled workers	21	4.2
Specialized social, legal, teaching professionals	156	31.2
Specialized health, science, engineering, business professionals	111	22.2
Managers	31	6.2
Senior managers	2	.4
**Work in the health sector**		
Yes	172	34.4
No	328	65.6
**Single residence**		
Individual	35	7.0
With others	465	93.0
**Presence of elderly people in the family**		
Yes	259	51.8
No	241	48.2
**Under chronic diseases**		
Diseases	134	26.8
No diseases	366	73.2
**Smoking**		
Yes	57	11.4
No	443	88.6
**With disability**		
Yes	11	2.2
No	489	97.8
**Diagnosis psychiatric disorder**		
Yes	21	4.2
No	479	95.8

### Tools

The following tools were used to collect data for each subject’s demographic characteristics, past medical history, and medication history together with the following tools:

1. *State-Trait Anxiety Inventory* (*STAI*) [25, 30]. This tool has been used extensively in research and clinical practice. It comprises separate self-report scales for measuring state and trait anxiety. The S-Anxiety Inventory (STAI Form Y-1) consists of 20 statements that evaluate how respondents feel “right now, at this moment.” It includes feelings of apprehension, tension, nervousness, and worry. We only used the State-Anxiety inventory in this study.
2. *COVID-19 Life Events-Anxiety Inventory* (*C-19LAI*). This inventory comprises 40 items divided in two scales (C-19L and C-19A). The first scale includes a set of 20 items and was developed from data obtained from a pilot study (33 participants, males and females, 18 years old and above). We rewrote and clarified items and words.

The respondents selected recent stressors (life events) related with COVID-19 as being personally diagnosed with coronavirus infection or having a family member or a friend diagnosed with COVID-19, death of a family member or a friend, exhaustion due to quarantine, health precautions and instructions related to COVID-19 pandemic, fear of getting medical consultation and of course financial, educational, family, and marriage problems that takes place as a consequence to the current pandemic. In addition, subjects responded to the 20 anxiety-related statements using a four-point rating scale ranging from “not at all” to “fairly often” regardless whether the problem or life event occurred.

Our rationale for developing the C-19A inventory is that it provides a self-report scale for measuring anxiety that is specifically related to COVID-19 in comparison to general anxiety (as in Spielberger [25]) and evaluates how respondents feel in the present moment.

This battery was designed by the first and fourth author.

### Procedure

The first phase of the study involved designing a tool specifically to record life events and anxiety during COVID-19 pandemics, and based on a review of domestic and foreign literature. Four existing relevant scales were identified: Fear of COVID-19 Scale [31], COVID Stress Scales [22], COVID-19 Psychological Destruction Scale [32], and the State-Trait Anxiety Inventory [25, 30]. However, our review found no scale that could capture events and related anxiety with regard to COVID-19 in the Egyptian cultural context.

For this phase, a pilot study was conducted online using a convenience sample of 33 Egyptian youth, adults, and elderly. The pilot was conducted from April 4 to 10, 2020, and recruited 7 (21.1%) males and 26 (78%) females, where the majority of the sample (27.3%) are less than 20, (39.4%) are within the age range from 21-40 and (33.3%) are more than 41 years. Five participants (15.2%) had less than 12 years and 28 had more than 12 (84.8%) years of education. Sixteen (48.5%) participants were single, 13 (39.4%) were married, 1 was widowed, and 3 were separated/divorced. The vast majority (31) was from urban areas while two were rural. Six participants were from Cairo, 12 from Giza, 14 from Gharbia, and 1 from Ismailia. The occupations of participants in the pilot study were varied.

Participants were presented with open-ended questions as to the major stressors in their lives brought by COVID-19 and the psychological effects this has had on them.

Based on the data obtained from this pilot study and in line with the theoretical framework, a 40-item inventory was developed that used two scales: C-19L and C-19A. The first scale is used for a set of 20 items that prompt respondents to select recent stressors (life events) related with COVID-19 and rate their anxiety on 20 anxiety on four-point scale ranging from “not at all” to “fairly often” regardless of whether the problem or life event occurred. The item pool was revised and items that were nonspecific, redundant, or too infrequent or complex were removed. Finally, a language specialist was consulted and suggested corrections were made. The new tool was applied to a sample of 500 participants aged 18–50 years through online surveys together with the State-Trait Anxiety Inventory (STAI) [25] as a gold standard tool for comparison.

### Statistical analysis

Collected data were coded, tabulated, and statistically analyzed using IBM SPSS V22. Quantitative data were described using minimum and maximum of the range as well as mean±SD (standard deviation) and compared using independent *t* test. Qualitative data were described using number and percentage, and compared using chi-square test. ROC curve was used to evaluate the performance of different tests differentiate between certain groups. The level of significance was taken at *p* < 0.050 and otherwise non-significant.

Diagnostic characteristics were calculated as follows:

- Sensitivity = (True positive test/Total positive golden) × 100
- Specificity = (True negative test/Total negative golden) × 100
- Diagnostic accuracy = ([True positive test + True negative test]/Total cases) × 100
- Youden’s index = sensitivity + specificity−1
- Predictive positive value = (True positive test/Total positive test) × 100
- Predictive negative value = (True negative test/Total negative test) × 100
- LR+ = (sensitivity/1− specificity)
- LR− = (1− sensitivity/specificity)
- LR= LR+/LR−
- Kappa = Observed agreement−chance agreement/1−chance agreement

## Results

The most frequent age group (*n*=260, 52.0%) was < 40 years followed by 40−59 years (*n*=170, 34.0%) and ≥ 60 years (*n*=70, 14.0%). Males comprised about one-third of cases (*n*=182, 36.4%). Table 1 shows the demographic characteristics of the participants.

Those aged 40 to 59 years were significantly more prominent in reporting COVID-19-related anxiety while those under 40 years were the least frequent in reporting such anxiety and the cases of those ≥ 60 years were non-significantly different. Twenty-nine subjects (9 males, 20 females) were infected with COVID-19. Of our sample, 30 subjects had a family member or a relative infected with COVID-19 and 34 subjects (10 males, 22 females) experienced death of a relative or a friend due to COVID-19.

No significant difference in anxiety was observed between genders. The State-Trait Anxiety Inventory (STAI) [25] (mean±SD) score is 50.3±7.4 with range 30.0−81.0, indicating anxiety in 473 (94.6%) respondents. Table 2 shows the age and gender of all participants according to State Trait Anxiety Inventory.

**Table 2:** Age and gender in all participants according to State Trait Anxiety Inventory (STAI) [25]

Variables		All cases (***N***=500)	Anxiety (***N***=473)	No anxiety (***N***=27)	***p*** value^#^
**Age (years)**	**< 40.0**	260 (52.0%)	240 (50.7%)a	20 (74.1%)b	0.029*
	**40.0−59.0**	170 (34.0%)	167 (35.3%)a	3 (11.1%)b	
	**≥ 60.0**	70 (14.0%)	66 (14.0%)a	4 (14.8%)a	
**Gender**	**Male**	182 (36.4%)	174 (36.8%)	8 (29.6%)	0.452
	**Female**	318 (63.6%)	299 (63.2%)	19 (70.4%)	

#Chi square test

^*^ < 0.005 significant

On the other hand, on using COVID-19 Life Events-Anxiety Inventory, it was shown that the anxiety score was significantly higher in anxiety cases than non-anxiety cases among all cases, in both sexes and among different age groups except ≥ 60.0 years as shown in Table 3.

**Table 3:** Age and gender in all participants according to COVID-19 Life Events-Anxiety Inventory (C-19LAI)

Categories		Anxiety		No anxiety		***p*** value^^^
		***N***	Mean±SD	***N***	Mean±SD	
**Total cases**		473	57.4±14.7	27	38.4±13.1	<0.001*
**Gender**	**Male**	174	58.6±16.1	8	32.6±12.1	<0.001*
	**Female**	299	56.6±13.9	19	40.8±13.0	<0.001*
**Age (years)**	**< 40 years**	240	51.9±13.4	20	35.8±9.5	<0.001*
	**40-59 years**	167	64.0±13.6	3	29.7±11.9	<0.001*
	**> 60 years**	66	60.6±14.3	4	58.3±12.9	0.752

^Independent *t* test

^*^Significant

**Fig. 1:**
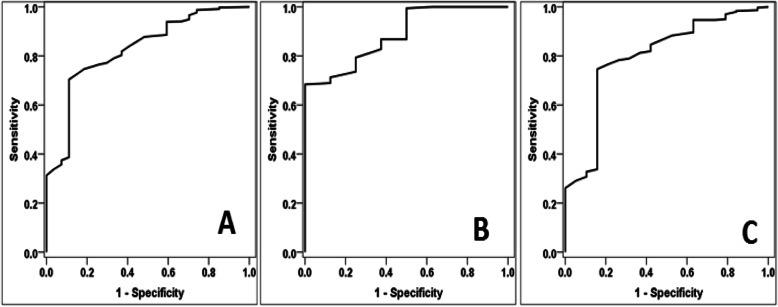
ROC curve for COVID-19 anxiety score in diagnosing anxiety [25] among all sample, males, and females

Our study also shows that the COVID-19 Anxiety Score had moderate diagnostic performance in diagnosing anxiety and was higher in females than in males. Diagnostic performance of the COVID-19 LAI suggested it had high specificity and PPV but low sensitivity and NPV. The characteristics were higher in males as shown in Table 4 and Fig. 1.

**Table 4:** Diagnostic performance and characteristics of COVID-19 Life Events—Anxiety Inventory

**Categories**		***N***	**AUC**	**SE**	***p*** **value**	**95% CI**	**Cut off**
**Total cases**		500	0.826	0.038	<0.001*	0.752−0.901	≥49.0
**Gender**	**Male**	182	0.886	0.047	<0.001*	0.795−0.977	≥49.0
	**Female**	318	0.797	0.054	<0.001*	0.691−0.902	≥47.0
**Diagnostic characteristics**							
**Characters**	**Male** ≥49.0		**Female** ≥47.0			**Both together** **Male** ≥49.0 **Female** ≥47.0	
	**Value**	**95% CI**	**Value**	**95% CI**		**Value**	**95% CI**
**Sensitivity**	68.4%	60.9%–75.2%	76.6%	71.4%–81.3%		73.6%	69.4%–77.5%
**Specificity**	100%	63.1%–100%	78.9%	54.4%–93.9%		85.2%	66.3%–95.8%
**DA**	69.8%	62.5%–76.4%	76.7%	71.7%–81.3%		74.2%	70.1%–78.0%
**YI**	68.4%	61.5%–75.3%	55.5%	36.6%–74.5%		58.8%	44.8%–72.7%
**PPV**	100%	96.9%–100%	98.3%	95.7%–99.5%		98.9%	97.1%–99.7%
**NPV**	12.7%	5.6%–23.5%	17.6%	10.2%–27.4%		15.5%	10.1%–22.4%
**LR+**	>100.0	>100–>100	3.64	1.52–8.71		4.97	2.01–12.29
**LR-**	0.32	0.25–0.39	0.30	0.22–0.40		0.31	0.25–0.39
**LR**	>100.0	>100–>100	12.27	3.94–38.17		16.01	5.43–47.20
**Kappa**	0.160	0.059–0.260	0.211	0.107–0.316		0.189	0.114–0.263

*AUC* area under curve, *SE* standard error, *CI* confidence interval, *YI* Youden’s index, *DA* diagnostic accuracy, *PPV* positive predictive value, *NPV* negative predictive value, *LR*+ positive likelihood ratio, *LR−* negative likelihood ratio, *LR* diagnostic odds ratio

*Significant

### Concurrent validity: correlation with the State-Trait Anxiety Scale

The life events scale correlates in a positive, moderate, and significant way (*p* ≤ 0.01) with the Spielberger Anxiety scale (*r* = 0.289). The COVID-19 anxiety scale correlates in a positive, moderate, and significant way (*p* ≤ 0.01) with the Spielberger Anxiety scale (*r* = 0.407).

### Concurrent validity: correlation with the State-Trait Anxiety Scale

The life events scale correlates in a positive, moderate, and significant way (*p* ≤ 0.01) with the Spielberger Anxiety scale (*r* = 0.289).

The COVID-19 anxiety scale correlates in a positive, moderate, and significant way (*p* ≤ 0.01) with the Spielberger Anxiety scale (*r* = 0.407).

### Reliability analysis

A reliability analysis comprising 20 items was carried out on the life events scale (Table 2). Cronbach’s alpha showed that the questionnaire reaches the acceptable reliability (*α* = 0.815). Most items appeared to be worthy of retention, resulting in a decrease in the alpha if deleted. The exceptions to this were items Q1, Q2, Q3, and Q17, which would increase the alpha to *α* = 0.818. Thus, these items were deleted in order to verify the validity of the internal consistency between the questionnaire items. Table 2 indicates that all items have roughly equivalent means and standard deviations within the COVID-19 life events except Q1, Q2, Q3, and Q17. Q1 (Mean = .06, SD = .234), Q2 (Mean = .06, SD = .238), Q3 (Mean = .07, SD = .252), and Q17 (Mean = .03, SD = .159) have lower mean values compared with the other items in life events section of C-19LAI. It is shown that Cronbach’s alpha of internal consistency reliability is 0.815. Most items appeared to be worthy of retention, resulting in a decrease in the alpha if deleted and each value of item-total correlation was in the range 0.095–0.607. The highest correlations were noticed in Q1 = 0.607 and the lowest in Q9 = 0.095. It can be seen that all items have roughly equivalent means and standard deviations within the COVID-19 life events scale as shown in Table 5.

**Table 5:** Mean, standard deviation (SD), Cronbach’s alpha, and item-total correlation of each item (C-19LAI)

Items	Mean	SD	Cronbach’s alpha^**b**^	Item-total correlation
**LE**	9.41	4.06	.815	
Q1	.06	.234	.817	.101
Q2	.06	.238	.818	.095
Q3	.07	.252	.817	.119
Q4	.32	.467	.815	.219^a^
Q5	.33	.471	.817	.208^a^
Q6	.49	.500	.802	.470^a^
Q7	.55	.498	.803	.454^a^
Q8	.39	.488	.804	.432^a^
Q9	.57	.496	.805	.416^a^
Q10	.80	.402	.804	.442^a^
Q11	.72	.447	.800	.513^a^
Q12	.59	.493	.799	.514^a^
Q13	.53	.499	.800	.498^a^
Q14	.84	.367	.807	.381^a^
Q15	.70	.459	.798	.546^a^
Q16	.78	.416	.806	.402^a^
Q17	.03	.159	.816	.131
Q18	.56	.497	.808	.375^a^
Q19	.60	.491	.793	.607^a^
Q20	.44	.496	.805	.425^a^

^a^Correlation is significant at the 0.01 level

^b^Cronbach’s alpha value if an item is deleted

It is also shown in Table 6 that Cronbach’s alpha of internal consistency reliability is 0.947. Most items appeared to be worthy of retention, resulting in a decrease in the alpha if deleted, and each value of item-total correlation was in the range 0.566–0.769. The highest correlations were noticed in Q1 = 0.769 and the lowest in Q9 = 0.566. It can be seen that all items have roughly equivalent means and standard deviations within the COVID-19 anxiety scale.

**Table 6:** Mean, standard deviation (SD), Cronbach’s alpha, and item-total correlation of each item in C-19LAI

Items	Mean	SD	Cronbach’s alpha^**b**^	Item-total correlation
**AC-19**	56.34	15.26	.947	
Q1	2.99	1.030	.943	.769^a^
Q2	3.25	.959	.945	.643^a^
Q3	3.24	1.007	.944	.671^a^
Q4	2.89	1.069	.943	.764^a^
Q5	2.81	1.085	.943	.736^a^
Q6	2.86	1.114	.944	.703^a^
Q7	2.80	1.165	.945	.617^a^
Q8	2.42	1.181	.944	.707^a^
Q9	2.63	1.210	.946	.566^a^
Q10	2.92	1.070	.944	.697^a^
Q11	2.87	1.106	.944	.720^a^
Q12	2.89	1.090	.944	.722^a^
Q13	2.66	1.164	.944	.709^a^
Q14	3.46	.831	.946	.559^a^
Q15	3.09	.952	.944	.720^a^
Q16	3.15	.967	.944	.678^a^
Q17	1.63	1.015	.949	.374^a^
Q18	2.67	1.174	.945	.635^a^
Q19	2.73	1.131	.943	.777^a^
Q20	2.37	1.204	.945	.635^a^

^a^Correlation is significant at the 0.01 level

^b^Cronbach’s alpha value if an item is deleted

## Discussion

Unfortunately, little attention has been given to designing an instrument to measure mental health symptoms, especially anxiety, during the COVID-19 pandemic and particularly in the Arabic-speaking Egyptian population. In this respect, this study was conducted to develop a valid and reliable tool to measure the different life events and the degree of anxiety related to it during the COVID-19 pandemic. The COVID-19 Life Events-Anxiety Inventory (C-19LAI) scale is the first Arabic-designed tool to measure COVID-19-related life events and anxiety related. Among a sample of 500 subjects, 94.6% reported significant anxiety as measured using the State-Trait Anxiety Inventory (STAI) [25] in the early stages of the coronavirus pandemic in Egypt, which is much higher than the 53.5% reported in previous studies in Egypt by Arafa et al. [33], which might be due to the different timing of the study. The earlier study was conducted during the very early phases of the pandemic and included much larger sample of 1629 subjects. In this study, the pandemic was peaking; hence, constant exposure to the news about worldwide fatalities or infection rates of the pandemic has led individuals to experience fear, anxiety, and depression [34].

The results of this study support the COVID-19 Life Events-Anxiety Inventory (C-19LAI) scale as a useful anxiety scale for its diagnostic qualities, sensitivity (73.6%), specificity (85.2%), and it is comparable especially in terms of specificity to other psychiatric screening tools designed during the COVID-19 pandemic as a coronavirus anxiety-related scale [29], which has 90% sensitivity and 85% specificity. In addition, the sensitivity (89%) and specificity (82%) values for the Generalized Anxiety Disorder 7 (GAD-7), a popular measure of anxiety disorder symptoms, are slightly below those of the CAS [35]. Relatedly, the sensitivity (73%) and specificity (74%) values for the State-Trait Inventory for Cognitive and Somatic Anxiety (STICSA), another measure of anxiety, also fall below those of the CAS [36]. In terms of a general psychiatric screener, the sensitivity (77%) and specificity (71%) values for the General Health Questionnaire (GHQ), a measure extensively used in primary care research to assess depression, anxiety, somatic concomitants, and social impairment, also fall below those of the CAS [37].

It can be seen that the COVID-19 Life Events-Anxiety Inventory (C-19LAI) scale has a positive, moderate, and significant correlation (*p* ≤ 0.01) with the Spielberger Anxiety scale (*r* = 0.289 and *r* = 0.407, respectively). Furthermore, the COVID-19 anxiety score was significantly higher in anxiety cases than in non-anxiety cases among all cases on comparison with the State-Trait Anxiety Inventory (STAI) scores for males and females and among different age groups except ≥ 60.0 years, which gives us a broad idea about its concurrent validity.

The first part of the scale, which related to different life events related to the COVID-19 pandemic, reached acceptable reliability (*α* = 0.815); in the second part, the COVID-19 anxiety scale showed an internal consistency reliability was 0.947, which is comparable to the coronavirus anxiety-related scale [29] with reliability of *α* = 0.93.

Although our study has the strength of a robust sample size and wide range of ages from different cities in Egypt, it is important to consider some limitations. First, no factor analysis was done after the pilot study. Second, a convenience sample was used that does not adequately represent the Egyptian population, which reduces generalizability. Third, the cross sectional design does not reveal causality between the COVID 19 pandemic and anxiety. Fourth, we did not investigate if any of our subjects are on or received any psychiatric treatment and we had not investigated if the subjects had. Hence, future studies should consider a longitudinal design. It is also worth mentioning that the stability of the scale over time was also not studied.

## Conclusions

The COVID-19 Life Events-Anxiety Inventory (C-19LAI) scale is a reliable and valid scale that can measure anxiety and events related to anxiety during the COVID 19 pandemic.

### Recommendation

Future study is needed on the application of our tool on psychiatric patients.

### Supplementary Information

**Additional file 1.**

## Data Availability

The datasets used and/or analyzed during the current study are available from the corresponding author on reasonable request.

## Appendix

Corresponding Author regarding the Arabic and English version of this inventory (C-19LAI)

Omaima Refat Elsayed Madkor, The Martyr Engineer Ahmed Shawky Hospital for Elderly Medicine, Ain Shams University Hospitals, Abbasiya, 1156, Cairo, Egypt. Tel: +01115047862, Email: OmaimaMadkor@med.asu.edu.eg

The (C-19LAI) Arabic and English version Copyright Owner

## Abbreviations

COVID-19: Coronavirus pandemic 2019
C-19LAI: COVID-19 Life Events-Anxiety Inventory
WHO: World Health Organization
STAI: State-Trait Anxiety Inventory
CAS: Coronavirus Anxiety Scale
T-Anxiety: Trait anxiety
S-Anxiety: State anxiety
DA: Diagnostic accuracy
PPV: Predictive positive value
NPV: Predictive negative value
LR: Likelihood ratio
Y: Youden’s index
ROC: Receiver operator characteristics curve
STICSA: State-Trait Inventory for Cognitive and Somatic Anxiety
GAD-7: Generalized Anxiety Disorder 7
GHQ: General Health Questionnaire
