# Supplementary material for: COVID-19 Life Events-Anxiety Inventory (C-19LAI): development, reliability, and validity study on Egyptian population
Source: Middle East Curr Psychiatry. 2021 Apr 2;28(1):21. doi: 10.1186/s43045-021-00101-z (PMC8016502; doi:10.1186/s43045-021-00101-z)
Supplement: Supplementary file 1 — Additional file 1. [file 43045_2021_101_MOESM1_ESM.docx]

| **بطارية كوفيد-19 أحداث الحياة والقلق النسخة العربية (مصر)** 2020 |
| --- |
| C-19LAI - Madkor *et al*. 2020 |

**فيما يلي بعض المشكلات المهمة والمواقف المثيرة للشدة المتعلقة بظهور وباء كورونا، يرجى اختيار ما يناسبك مما يلي:**

1. **الإصابة الشخصية بفيروس كورونا**

| - **حدثت المشكلة** | | | | | |
| --- | --- | --- | --- | --- | --- |
| - **لم تحدث المشكلة** | | | | | |
| - **حدد درجة قلقك في هذه اللحظة من المشكلة رقم (1) سواء حدثت أو لم تحدث** | | | | | |
|  | **1** | **2** | **3** | **4** |  |
| **غير قلق مطلقاً** |  |  |  |  | **قلق جداً** |

1. **إصابة أحد أفراد أسرتك أو المقربين بفيروس كورونا**

| - **حدثت المشكلة** | | | | | |
| --- | --- | --- | --- | --- | --- |
| - **لم تحدث المشكلة** | | | | | |
| - **حدد درجة قلقك في هذه اللحظة من المشكلة رقم (2) سواء حدثت أو لم تحدث** | | | | | |
|  | **1** | **2** | **3** | **4** |  |
| **غير قلق مطلقاً** |  |  |  |  | **قلق جداً** |

1. **موت قريب أو صديق بسبب فيروس كورونا**

| - **حدثت المشكلة** | | | | | |
| --- | --- | --- | --- | --- | --- |
| - **لم تحدث المشكلة** | | | | | |
| - **حدد درجة قلقك في هذه اللحظة من المشكلة رقم (3) سواء حدثت أو لم تحدث** | | | | | |
|  | **1** | **2** | **3** | **4** |  |
| **غير قلق مطلقاً** |  |  |  |  | **قلق جداً** |

1. **الإنهاك بسبب الالتزام باتباع الإرشادات الصحية**

| - **حدثت المشكلة** | | | | | |
| --- | --- | --- | --- | --- | --- |
| - **لم تحدث المشكلة** | | | | | |
| - **حدد درجة قلقك في هذه اللحظة من المشكلة رقم (4) سواء حدثت أو لم تحدث** | | | | | |
|  | **1** | **2** | **3** | **4** |  |
| **غير قلق مطلقاً** |  |  |  |  | **قلق جداً** |

1. **صعوبة الحصول على الاستشارات الطبية للأمراض الأخرى**

| - **حدثت المشكلة** | | | | | |
| --- | --- | --- | --- | --- | --- |
| - **لم تحدث المشكلة** | | | | | |
| - **حدد درجة قلقك في هذه اللحظة من المشكلة رقم (5) سواء حدثت أو لم تحدث** | | | | | |
|  | **1** | **2** | **3** | **4** |  |
| **غير قلق مطلقاً** |  |  |  |  | **قلق جداً** |

**جميع الحقوق محفوظة للمؤلفين © أميمة مدكور، بسنت عبدالجواد 2020**

**يحظر الاستنساخ غير المصرح به من هذه البطارية بدون إذن المؤلفين** [**OmaimaMadkor@med.asu.edu.eg**](mailto:OmaimaMadkor@med.asu.edu.eg) **1**

1. **مشكلات مادية**

| - **حدثت المشكلة** | | | | | |
| --- | --- | --- | --- | --- | --- |
| - **لم تحدث المشكلة** | | | | | |
| - **حدد درجة قلقك في هذه اللحظة من المشكلة رقم (6) سواء حدثت أو لم تحدث** | | | | | |
|  | **1** | **2** | **3** | **4** |  |
| **غير قلق مطلقاً** |  |  |  |  | **قلق جداً** |

1. **مشكلات متعلقة بالعمل**

| - **حدثت المشكلة** | | | | | |
| --- | --- | --- | --- | --- | --- |
| - **لم تحدث المشكلة** | | | | | |
| - **حدد درجة قلقك في هذه اللحظة من المشكلة رقم (7) سواء حدثت أو لم تحدث** | | | | | |
|  | **1** | **2** | **3** | **4** |  |
| **غير قلق مطلقاً** |  |  |  |  | **قلق جداً** |

1. **الخلافات الأسرية أو الزواجية / الشجار مع الأطفال**

| - **حدثت المشكلة** | | | | | |
| --- | --- | --- | --- | --- | --- |
| - **لم تحدث المشكلة** | | | | | |
| - **حدد درجة قلقك في هذه اللحظة من المشكلة رقم (8) سواء حدثت أو لم تحدث** | | | | | |
|  | **1** | **2** | **3** | **4** |  |
| **غير قلق مطلقاً** |  |  |  |  | **قلق جداً** |

1. **مشكلات متعلقة بالتعليم**

| - **حدثت المشكلة** | | | | | |
| --- | --- | --- | --- | --- | --- |
| - **لم تحدث المشكلة** | | | | | |
| - **حدد درجة قلقك في هذه اللحظة من المشكلة رقم (9) سواء حدثت أو لم تحدث** | | | | | |
|  | **1** | **2** | **3** | **4** |  |
| **غير قلق مطلقاً** |  |  |  |  | **قلق جداً** |

1. **تغير الروتين اليومي/ تغيير خطط السفر وغيرها**

| - **حدثت المشكلة** | | | | | |
| --- | --- | --- | --- | --- | --- |
| - **لم تحدث المشكلة** | | | | | |
| - **حدد درجة قلقك في هذه اللحظة من المشكلة رقم (10) سواء حدثت أو لم تحدث** | | | | | |
|  | **1** | **2** | **3** | **4** |  |
| **غير قلق مطلقاً** |  |  |  |  | **قلق جداً** |

1. **البقاء في المنزل**

| - **حدثت المشكلة** | | | | | |
| --- | --- | --- | --- | --- | --- |
| - **لم تحدث المشكلة** | | | | | |
| - **حدد درجة قلقك في هذه اللحظة من المشكلة رقم (11) سواء حدثت أو لم تحدث** | | | | | |
|  | **1** | **2** | **3** | **4** |  |
| **غير قلق مطلقاً** |  |  |  |  | **قلق جداً** |

**جميع الحقوق محفوظة للمؤلفين © أميمة مدكور، بسنت عبدالجواد 2020**

**يحظر الاستنساخ غير المصرح به من هذه البطارية بدون إذن المؤلفين** [**OmaimaMadkor@med.asu.edu.eg**](mailto:OmaimaMadkor@med.asu.edu.eg) **2**

1. **المتابعة المفرطة لأخبار الوباء**

| - **حدثت المشكلة** | | | | | |
| --- | --- | --- | --- | --- | --- |
| - **لم تحدث المشكلة** | | | | | |
| - **حدد درجة قلقك في هذه اللحظة من المشكلة رقم (12) سواء حدثت أو لم تحدث** | | | | | |
|  | **1** | **2** | **3** | **4** |  |
| **غير قلق مطلقاً** |  |  |  |  | **قلق جداً** |

1. **الشعور بالوحدة أو العزلة**

| - **حدثت المشكلة** | | | | | |
| --- | --- | --- | --- | --- | --- |
| - **لم تحدث المشكلة** | | | | | |
| - **حدد درجة قلقك في هذه اللحظة من المشكلة رقم (13) سواء حدثت أو لم تحدث** | | | | | |
|  | **1** | **2** | **3** | **4** |  |
| **غير قلق مطلقاً** |  |  |  |  | **قلق جداً** |

1. **عدم وعي الآخرين بخطورة الموقف**

| - **حدثت المشكلة** | | | | | |
| --- | --- | --- | --- | --- | --- |
| - **لم تحدث المشكلة** | | | | | |
| - **حدد درجة قلقك في هذه اللحظة من المشكلة رقم (14) سواء حدثت أو لم تحدث** | | | | | |
|  | **1** | **2** | **3** | **4** |  |
| **غير قلق مطلقاً** |  |  |  |  | **قلق جداً** |

1. **الخوف من التعامل مع الآخرين**

| - **حدثت المشكلة** | | | | | |
| --- | --- | --- | --- | --- | --- |
| - **لم تحدث المشكلة** | | | | | |
| - **حدد درجة قلقك في هذه اللحظة من المشكلة رقم (15) سواء حدثت أو لم تحدث** | | | | | |
|  | **1** | **2** | **3** | **4** |  |
| **غير قلق مطلقاً** |  |  |  |  | **قلق جداً** |

1. **البعد عن رؤية الأهل والأصدقاء**

| - **حدثت المشكلة** | | | | | |
| --- | --- | --- | --- | --- | --- |
| - **لم تحدث المشكلة** | | | | | |
| - **حدد درجة قلقك في هذه اللحظة من المشكلة رقم (16) سواء حدثت أو لم تحدث** | | | | | |
|  | **1** | **2** | **3** | **4** |  |
| **غير قلق مطلقاً** |  |  |  |  | **قلق جداً** |

1. **الطلاق**

| - **حدثت المشكلة** | | | | | |
| --- | --- | --- | --- | --- | --- |
| - **لم تحدث المشكلة** | | | | | |
| - **حدد درجة قلقك في هذه اللحظة من المشكلة رقم (17) سواء حدثت أو لم تحدث** | | | | | |
|  | **1** | **2** | **3** | **4** |  |
| **غير قلق مطلقاً** |  |  |  |  | **قلق جداً** |

**جميع الحقوق محفوظة للمؤلفين © أميمة مدكور، بسنت عبدالجواد 2020**

**يحظر الاستنساخ غير المصرح به من هذه البطارية بدون إذن المؤلفين** [**OmaimaMadkor@med.asu.edu.eg**](mailto:OmaimaMadkor@med.asu.edu.eg) **3**

1. **تأثر صحتي الجسمية/ زيادة الوزن بسبب البقاء في المنزل**

| - **حدثت المشكلة** | | | | | |
| --- | --- | --- | --- | --- | --- |
| - **لم تحدث المشكلة** | | | | | |
| - **حدد درجة قلقك في هذه اللحظة من المشكلة رقم (18) سواء حدثت أو لم تحدث** | | | | | |
|  | **1** | **2** | **3** | **4** |  |
| **غير قلق مطلقاً** |  |  |  |  | **قلق جداً** |

1. **تأثر صحتي النفسية**

| - **حدثت المشكلة** | | | | | |
| --- | --- | --- | --- | --- | --- |
| - **لم تحدث المشكلة** | | | | | |
| - **حدد درجة قلقك في هذه اللحظة من المشكلة رقم (19) سواء حدثت أو لم تحدث** | | | | | |
|  | **1** | **2** | **3** | **4** |  |
| **غير قلق مطلقاً** |  |  |  |  | **قلق جداً** |

1. **التوقف عن القيام بالعمل التطوعي**

| - **حدثت المشكلة** | | | | | |
| --- | --- | --- | --- | --- | --- |
| - **لم تحدث المشكلة** | | | | | |
| - **حدد درجة قلقك في هذه اللحظة من المشكلة رقم (20) سواء حدثت أو لم تحدث** | | | | | |
|  | **1** | **2** | **3** | **4** |  |
| **غير قلق مطلقاً** |  |  |  |  | **قلق جداً** |

**جميع الحقوق محفوظة للمؤلفين © أميمة مدكور، بسنت عبدالجواد 2020**

**يحظر الاستنساخ غير المصرح به من هذه البطارية بدون إذن المؤلفين** [**OmaimaMadkor@med.asu.edu.eg**](mailto:OmaimaMadkor@med.asu.edu.eg) **4**
